# Expected and unexpected roles for dynein regulation of dendritic late endosomes

**DOI:** 10.1080/27694127.2022.2142888

**Published:** 2022-11-08

**Authors:** Chan Choo Yap, Bettina Winckler

**Affiliations:** Department of Cell Biology, University of Virginia, 1340 Jefferson Park Avenue, Pinn Hall 3226, Charlottesville, VA, USA

**Keywords:** Degradation, dendrite, dynein, late endosome, lysosome, maturation, neuron, NSG2, RAB7, RILP

## Abstract

Dendrites differ from axons in multiple ways, including the presence of minus-end out microtubules intermixed with the more conventional plus-end out microtubules. The mixed microtubule polarity makes regulation of directional transport in dendrites a challenge. Dynein can in principle be a retrograde and anterograde motor in dendrites. We show in our recent paper that dynein supports bi-directional transport of late endosomes in dendrites. We also show that overexpression of the RAB7 effector RILP which recruits dynein to late endosomes imparts retrograde bias onto late endosomes. Inhibition of dynein leads to a decrease in bi-directional motility of late endosomes, an expected result. Unexpectedly, inhibition of dynein also impairs endosome maturation as evidenced by increased association of GTP-RAB7 with late endosomes. Ultimately, dynein inhibition causes degradation defects of short-lived dendritic receptors and stunted dendrite morphologies. Much more work is required to fully understand how endosomal pathways are regulated in time and space in dendrites. Given the prevalence of neurological disorders where endosome-lysosome functions are impaired, this is a topic of great translational relevance.

**Abbreviations**: RILP: Rab-interacting lysosomal protein; EE: early endosome; LE: late endosome; lys: lysosome; NSG2: neural-specific gene 2; RILP-CT: RILP C-terminal truncation.

Transport of endocytosed cargos to their final cellular destinations require maturation of endosomes in a sequential and regulated fashion. Cargos enter through endocytosis into early endosomes (EEs) from where sorting to either recycling or degradation pathways take place. The decision to degrade is regulated by the small GTPase RAB7, which is the master regulator of EE-late endosome (LE) maturation and dynamics. Recruitment of RAB7-GTP initiates maturation of EEs (EEA1 and RAB5 positive) to LEs (RAB7 positive), which then carry degradative cargos to lysosomes where they undergo fusion. The properties of endosomes thus change as they mature, but the molecular mechanisms driving these sequentially coordinated maturation events are still not fully elucidated. Interference with RAB7 function leads to impaired maturation of EEs into LEs and to inhibition of degradation in mammalian cells. Lysosomes are the major cellular degradative compartment for cargos that enter the cell through endocytosis or macroautophagy/autophagy. Lysosomes tend to cluster at the perinuclear region near centrosomes. Linking LEs to microtubule motors is thus also required for degradation because cargos need to be moved from the periphery to pericentriolar lysosomes. Motor recruitment to LEs is regulated by RAB7.

Neurons are very large cells, and spatial regulation of membrane traffic, including transport to lysosomes for degradation, is more challenging than in smaller cell types. In neurons, the density of lysosomes in neurons decreases rapidly with increasing distance from the soma, so bulk degradation of membrane proteins occurs predominantly in the soma and proximal dendrites (Figure 1A). In axons, dynein is solely responsible for long-range retrograde transport of cargos back to somatic lysosomes. This is the case because microtubule polarity in axons is almost completely plus-end-out allowing directional transport to be accomplished by kinesins (for anterograde transport) and dynein (for retrograde transport). In dendrites, the polarity of microtubules is mixed; thus, retrograde transport can in principle be mediated by either dynein or kinesin. Degradation of dendritic cargos requires RAB7-dependent transport to the soma, but how such directional transport in dendrites is accomplished is unclear.

**Figure 1.**
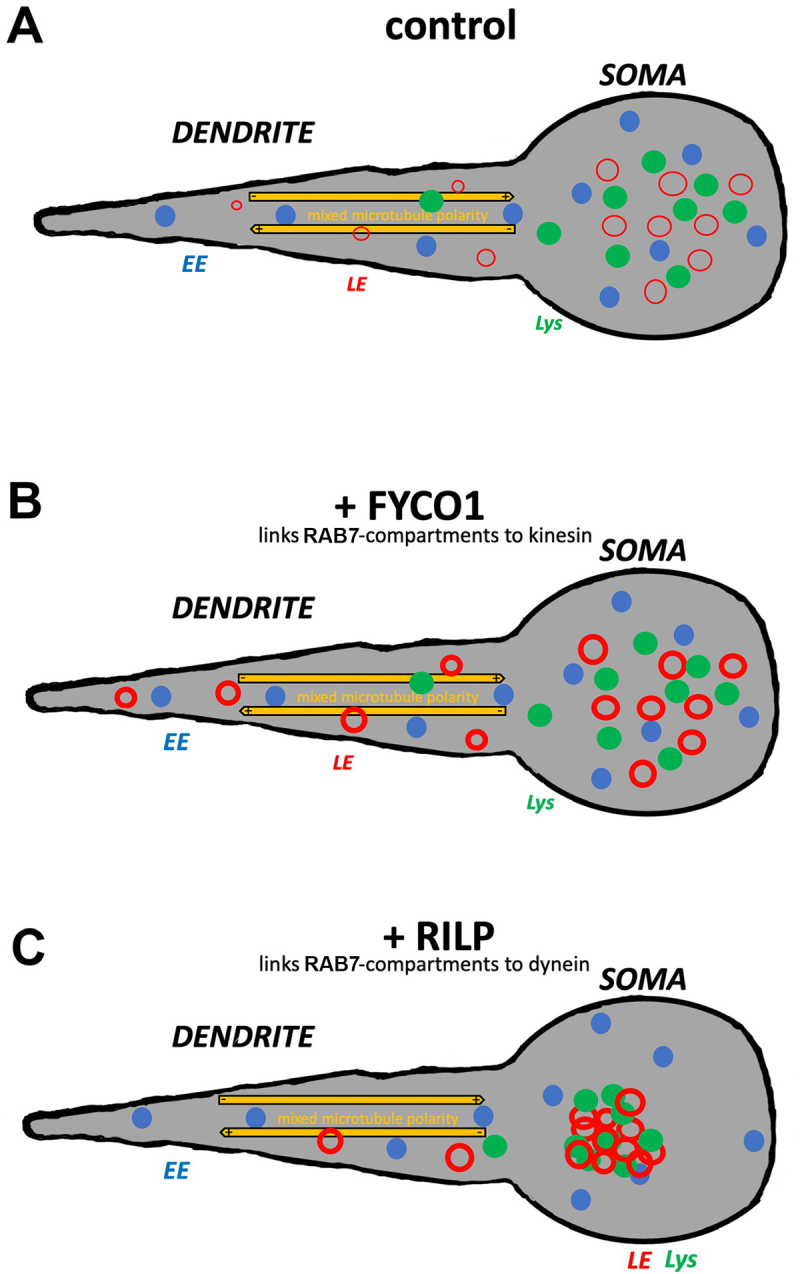
Effects of overexpression of RAB7-adaptors for kinesin and dynein on organelle positioning in dendrites. (**A**) Control: Steady-state organelle distribution in dendrites. (**B**) Overexpression of the kinesin adaptor FYCO1 leads to increased RAB7 accumulation on LEs (thick-rimmed red circles). (**C**) Overexpression of the dynein adaptor RILP leads to clustering of LEs and Lys in the perinuclear region and RAB7 accumulation (thick-rimmed red circles).

RAB7 exerts its functions via a multitude of effector proteins, which preferentially associate with activated RAB7. Among them are proteins that recruit microtubule motors to RAB7 compartments. In order to ask whether dynein or kinesin promote retrograde transport for RAB7-positive LEs in dendrites, we overexpressed two different RAB7 effectors, RILP (Rab interacting lysosomal protein) and FYCO1 (FYVE and coiled-coil domain autophagy adaptor 1), which link RAB7 LEs to dynein and kinesin, respectively [1]. We found that overexpressed FYCO1 colocalizes with RAB7 in a dispersed distribution along dendrites and in the soma (Figure 1B). In contrast, overexpression of RILP induces massive perinuclear clustering of RAB7 compartments which are also positive for RILP (Figure 1C). Somatic clustering of RAB7-positive compartments requires association of RILP with the dynein complex and with RAB7 because overexpression of either the N-terminus or C terminus of RILP (which fail to bind either RAB7 or the dynein complex, respectively) do not affect distribution of the compartments. Overexpression of RILP induces somatic clustering of all RAB7-positive compartments (LEs and lysosomes), but not of RAB7-negative compartments (Golgi, EEs, and M6PR-positive vesicles that shuttle between Golgi and endosomes). These results strongly suggest that dynein is the retrograde motor for RAB7-positive LEs in dendrites.

To test more directly if the dynein motor complex is involved in retrograde movement of dendritic LEs and successful delivery to somatic lysosomes, we inhibited dynein function in three ways (RILP C-terminal truncation [RILP-CT], CC1 domain of DCTN1/p150-glued, ciliobrevin). We assessed how inhibition of dynein affects degradation of a short-lived dendritic cargo (NSG2), using a cycloheximide pulse-chase approach, and motility of RAB7-LEs, using live imaging. Importantly, we found that recruitment of dynein to RAB7-LEs is crucial for efficient degradation of NSG2 (Figure 2).

**Figure 2.**
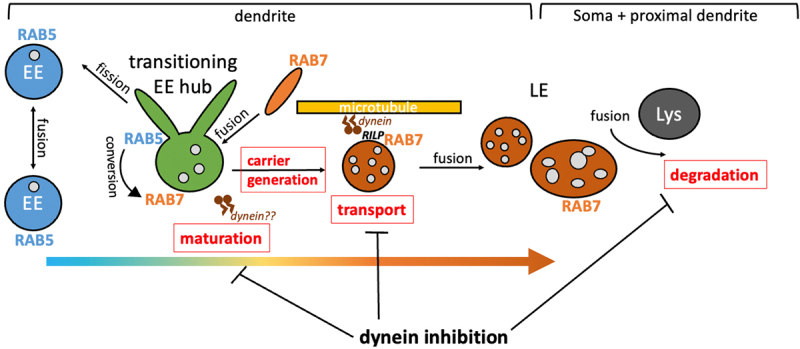
Effects of dynein inhibition on endosomal flux in dendrites. Inhibition of dynein inhibits transport of LEs, but also affects their maturation. Because maturation and transport are required steps upstream of degradation, dynein inhibition also impairs efficient degradation.

Analysis from our live imaging experiments revealed some expected and some unexpected features of dendritic LE transport. Expectedly, interfering with dynein function halts both the anterograde and retrograde movements of RAB7 and of the short-lived protein NSG1 (Figure 2). Unexpectedly, overexpression of full-length RILP preferentially boosts long distance retrograde movement of RAB7-LEs (Figure 3). These data suggest that dynein is able to promote long-range bidirectional transport of RAB7-LEs and of degradative cargos in dendrites. Retrograde directional bias is only observed when RILP is overexpressed. Surprisingly, very short distance motility seems independent of dynein.

**Figure 3.**
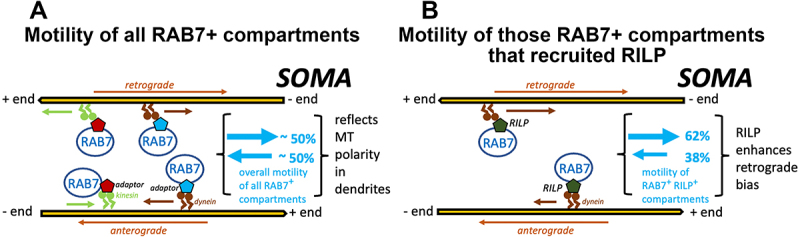
Motility of RAB7^+^ compartments in dendrites is retrogradely biased by RILP. (**A**) Live imaging of total RAB7^+^ compartments reveals close to 50:50 anterograde-retrograde motility found in dendrites. (**B**) Live imaging of dually positive RAB7^+^ and RILP^+^ compartments reveals a statistically significant retrograde bias.

A second unexpected finding is that overexpression of either RILP or the RILP-CT greatly increase RAB7 levels in neurons: Overexpressed RILP or RILP-CT lock RAB7 in its activated GTP form on endosomes (Figure 1C) and thereby prevent cycling of RAB7. RAB7 cycling between GDP- and GTP-bound states is thought to be important for temporal and spatial regulation of endosome maturation. Interestingly, overexpression of GFP-CC1 also increases RAB7 intensity on somatic endosomes. This observation led us to determine whether EE-LE maturation might be affected. EEA1 levels on dendritic endosomes are increased in neurons overexpressing RILP-CT but not GFP-CC1, raising the possibility of novel roles for dynein in endosome maturation (Figure 2). The profound disturbances in motility, degradation, and RAB7 cycling caused by increased WT RILP expression thus point to a delicate balancing of RAB7 activity and effector levels in neurons.

Looking into the future, we identify several open questions:

1. Is RILP an activating adaptor for dynein?
2. What is the molecular basis for the retrograde bias introduced by overexpression of RILP?
3. What is the mechanism by which dynein might facilitate endosome maturation?
4. How is sequential handover of RAB7 effectors regulated in time and space?
5. Which other motors (myosins or kinesins) also contribute to motility and affect positioning of LEs and other dendritic compartments, including lysosomes and autophagosomes?

In conclusion, our study demonstrates that interference with dynein recruitment to RAB7-LEs or with dynein function per se causes dendritic defects in endosome maturation, retrograde LE transport, and degradation. Much more work is required to fully understand how endosomal pathways are regulated in time and space in dendrites. Given the prevalence of neurological disorders where endosome-lysosome functions are impaired, this is a topic of great translational relevance.
